# Geographic Distribution of Ammonia-Oxidizing Archaea along the Kuril Islands in the Western Subarctic Pacific

**DOI:** 10.3389/fmicb.2017.01247

**Published:** 2017-06-30

**Authors:** Hongmei Jing, Shunyan Cheung, Xiaomin Xia, Koji Suzuki, Jun Nishioka, Hongbin Liu

**Affiliations:** ^1^CAS Key Laboratory for Experimental Study under Deep-sea Extreme Conditions, Institute of Deep-sea Science and Engineering, Chinese Academy of SciencesSanya, China; ^2^Division of Life Science, The Hong Kong University of Science and TechnologyKowloon, China; ^3^Faculty of Environmental Earth Science, Hokkaido UniversitySapporo, Japan; ^4^Institute of Low Temperature Science, Hokkaido UniversitySapporo, Japan

**Keywords:** AOA, *amoA*, pyrosequencing, geographic distribution

## Abstract

Community composition and abundance of ammonia-oxidizing archaea (AOA) in the ocean were affected by different physicochemical conditions, but their responses to physical barriers (such as a chain of islands) were largely unknown. In our study, geographic distribution of the AOA from the surface photic zone to the deep bathypelagic waters in the western subarctic Pacific adjacent to the Kuril Islands was investigated using pyrosequencing based on the ammonia monooxygenase subunit A (*amoA*) gene. Genotypes of clusters A and B dominated in the upper euphotic zone and the deep waters, respectively. Quantitative PCR assays revealed that the occurrence and ammonia-oxidizing activity of ammonia-oxidizing archaea (AOA) reached their maxima at the depth of 200 m, where a higher diversity and abundance of actively transcribed AOA was observed at the station located in the marginal sea exposed to more terrestrial input. Similar community composition of AOA observed at the two stations adjacent to the Kuril Islands maybe due to water exchange across the Bussol Strait. They distinct from the station located in the western subarctic gyre, where sub-cluster WCAII had a specific distribution in the surface water, and this sub-cluster seemed having a confined distribution in the western Pacific. Habitat-specific groupings of different WCB sub-clusters were observed reflecting the isolated microevolution existed in cluster WCB. The effect of the Kuril Islands on the phylogenetic composition of AOA between the Sea of Okhotsk and the western subarctic Pacific is not obvious, possibly because our sampling stations are near to the Bussol Strait, the main gateway through which water is exchanged between the Sea of Okhotsk and the Pacific. The vertical and horizontal distribution patterns of AOA communities among stations along the Kuril Islands were essentially determined by the *in situ* prevailing physicochemical gradients along the two dimensions.

## Introduction

Nitrification is a central process in the oceanic nitrogen cycle and can supply ∼25–36% of the N required by phytoplankton (Santoro et al., 2010). Ammonia oxidation as the first and rate-limiting step of nitrification is carried out by ammonia-oxidizing bacteria (AOB) and archaea (AOA) (Francis et al., 2005). AOA utilize ammonia as their major source of energy, catalyzing the conversion of ammonia to nitrite. The wide distribution and relative importance of AOA has been recognized after the isolation of *Nitrosopumilus maritimus* (Könneke et al., 2005) together with several metagenomic studies (Venter et al., 2004; Treusch et al., 2005). Compared with AOB, AOA have a potentially competitive advantage in nutrient-deficient marine ecosystems (Martens-Habbena et al., 2009), and are found more abundant in seawater (Mincer et al., 2007; Beman et al., 2008, 2010), therefore might be responsible for the most of the ammonia-oxidation occurring in the open ocean.

It has been reported previously that the abundance of archaea increases gradually in the cold deeper water along the oceanic vertical gradient (Karner et al., 2001) as a response to the hydrographic conditions. Similarly, vertically segregated groups of AOA have been described (Francis et al., 2005). For example, the “group A” or the “shallow” group (WCA) and “group B” or the “deep” group (WCB), primarily derived from the shallow euphotic zone (<200 m in depth) and deep waters (>200 m in depth), respectively, have been identified (Beman et al., 2008; Santoro et al., 2010; Mosier and Francis, 2011). WCA are actively involved in ammonia oxidation in the upper water column, but WCB express *amoA* throughout the dark ocean with only a fraction of them oxidizes ammonia (Hansman et al., 2009; Smith et al., 2016). Recently, AOA ecotypes HAC-AOA and LAC-AOA, which are adapted to high and low ammonia concentrations, respectively, have been identified. These ecotypes displayed distinct biogeographic and depth-related distribution patterns corresponding to the different ammonia concentrations (Sintes et al., 2013).

Ammonia-oxidizing archaea are widespread in the oceans (Francis et al., 2005; Herfort et al., 2007) and their distributions are influenced strongly by the associated physicochemical conditions (e.g., the depth of the water, the availability of substrate, and the light, salinity, and oxygen levels) (Francis et al., 2005; Beman and Francis, 2006; Agogué et al., 2008; Beman et al., 2008; Pouliot et al., 2009). The diversity (Pester et al., 2012) and community composition (Sintes et al., 2013) of AOA also varied geographically, for example, in different oceanic water masses along the latitudinal gradient in both the North Atlantic (Agogué et al., 2008) and Polar oceans (Galand et al., 2009; Kalanetra et al., 2009). It will be reasonable to assume that the community composition and abundance of AOA might vary in water masses separated by physical barriers (such as a chain of islands), and distinct distribution of AOA might be exhibited along the depth of the water columns.

We tested the hypothesis by using the high-throughput pyrosequencing of the ammonia monooxygenase gene subunit A (*amoA*) gene in samples collected from the Sea of Okhotsk and the western subarctic Pacific adjacent to the Bussol Strait of the Kuril Islands during summer 2014. The Sea of Okhotsk is one of the marginal seas of the North Pacific Ocean and is recognized as the most productive marine basin amongst the world’s oceans (Sorokin and Sorokin, 1999). It is reported that large amounts of dissolved/particulate organic carbon, and iron are transported from the Sea of Okhotsk to the subarctic Pacific Ocean via intermediate water transport through the deep passage of the Bussol Strait (i.e., with a sill depth of 2,300 m) (Nishioka et al., 2013; Sohrin et al., 2014). In addition, among the geographical studies on AOA communities conducted in recent years, only a few extended through the entire water column to the deep layer (Mincer et al., 2007; Yakimov et al., 2007; Agogué et al., 2008; De Corte et al., 2009; Beman et al., 2012; Sintes et al., 2013, 2016). Therefore, four different depths (from the surface photic zone to the deep bathypelagic waters) at each station were sampled in our study to provide a vertical profile of AOA along the steep gradients of light, temperature, salinity and N concentration. Our data demonstrated a clear geographic distribution of AOA along these vertical profiles at these different stations.

## Materials and Methods

### Sample Collection

**FIGURE 1:**
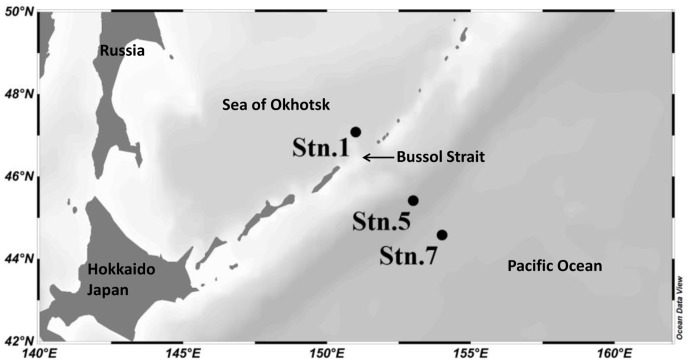
Location of the sampling stations in the Sea of Okhotsk and western subarctic Pacific.

Seawater samples were collected during June 2014 from three stations: Stn. 1, located at the southeast part of the Sea of Okhotsk deep basin; Stn. 5, located on the Pacific side of the Kuril Islands; and Stn. 7, located further away into the western subarctic gyre of the North Pacific Ocean (**Figure 1**). Stns 1 and 5 are both located near to the Bussol Strait where strong vertical mixing and the horizontal transport of water between the Sea of Okhotsk and the western subarctic Pacific take place. Water from four different depths, representing the surface (5 m), subsurface (200 m), mesopelagic (1000 m) and bathypelagic waters (3000 m), was collected using a CTD carousel water sampler with X-Niskin bottles (General Oceanics, Miami, FL, United States). Shortly following the collection of ∼2–3 L seawater, this was filtered onto a 2.0 μm and then 0.22 μm pore-size polycarbonate filters (47 mm, EMD Millipore, Billerica, MA, United States). The filters to be used for RNA analysis were immersed in RNAlater solution (Ambion, Thermo Fisher Scientific, Corp., Waltham, MA, United States) immediately after filtration. All the filters were then flash frozen and stored at -80°C until extraction on land.

The *in situ* environmental parameters (temperature, salinity, depth, and dissolved oxygen) were recorded with a CTD (General Oceanics, Miami, FL, United States). Samples for measuring inorganic nutrients (i.e., nitrate, nitrite, ammonia, phosphate, and silicate) were collected in acrylic tubes after filtered through 0.22 μm membranes, which were immediately stored at -20°C until they could be analyzed in the onboard laboratory. The concentrations of nutrients were measured with an auto-analyzer (QuAAtro, BLTEC. Co., Ltd), which was calibrated with certified seawater nutrient reference material (RM; KANSO).

### DNA and RNA Extraction and cDNA Synthesis

Genomic DNA was extracted from the 0.22 μm polycarbonate filters with a PureLink Genomic DNA Kit (Invitrogen, Thermo Fisher Scientific, Corp., Carlsbad, CA, United States), eluted into 100 μl Tris-EDTA (TE) buffer and stored at -80°C. Total RNA was extracted from the 0.22 μm polycarbonate filters with the TRIzol plus RNA purification kit (Invitrogen). RNAlater immersing the filters was removed before the preparation with TRIzol Reagent, and the extracted RNA was finally eluted in 50 μl of elution buffer. The concentrations of DNA and RNA were measured with a NanoDrop 2000 Spectrophotometer (Thermo Scientific, Thermo Fisher Scientific, Corp.).

Before cDNA synthesis, purified total RNA was treated with DNase I (Invitrogen) and incubated at room temperature for 15 min to eliminate any potential DNA contamination. The DNase I was then inactivated by heating at 65°C for 10 min with 25 mM EDTA. Total RNA (about 200 ng) was then reverse transcribed to cDNA with random hexamers using the SuperScript III first strand cDNA synthesis kit (Invitrogen) following the instruction on the first-strand synthesis with random primers. A parallel reaction without SuperScript III RT was used as an RT-PCR negative control. Synthesized cDNA was further digested with 2 U RNase H at 37°C for 20 min to remove residual RNA and then it was used for subsequent PCR amplification. Non-RT samples were always used as negative controls.

### Quantitative PCR

The abundance of the *amo*A gene and gene transcripts was determined by the StepOnePlus quantitative PCR (qPCR) system (Applied Biosystems, Inc., Carlsbad, CA, United States), with 25 μl of the SYBR^®^ Premix Ex Taq^TM^ kit (Takara Bio, Inc., Shiga, Japan), 0.3 μM of the Arch-amoAF/Arch-amoAR primer (Francis et al., 2005) and 2 μl of each DNA/cDNA as the template. The standard curve for absolute quantification was constructed using plasmid amplicons that were quantified on an Agilent 2100 bioanalyzer using DNA 7500 chips, according to the manufacturer’s protocol (Agilent Technologies, Inc., Santa Clara, CA, United States). Triplicate qPCR reactions were performed for each sample with efficiencies around 110%, and the gene copy number was normalized to the quantity of the gene and gene transcripts. The theoretical copy number was calculated to the size of the input PCR amplicon. In parallel, negative controls without reverse transcriptase and template were also prepared for the cDNA samples and no amplicons were produced. In addition, AOA group-specific assays for “shallow” water column ecotype A (WCA) and “deep” water column ecotype B (WCB) (Mosier and Francis, 2011) were conducted with efficiencies around 93% using the SYBR^®^ Premix Ex Taq^TM^ kit (Takara Bio, Inc.) (Santoro et al., 2013).

### 454 Pyrosequencing and Bioinformatics Analysis

For each genomic DNA sample, independent triplicates were extracted as templates to amplify the *amoA* gene, using Arch-*amoA*F and Arch-*amoA*R primers and following the PCR protocols previously described (Francis et al., 2005). For comparative purposes, the cDNA of samples collected at 200 m from stations 1, 5, and 7 (i.e., Stn1-200 m, Stn5-200 m, Stn7-200 m) was also amplified. In order to enable sample multiplexing during sequencing, barcodes were incorporated between the adapter and forward primer. Nuclease-free water was used as the negative control in each reaction. Triplicate PCRs were performed for each sample and the amplicons were pooled and subsequently purified with the illustra^TM^ GFX^TM^ PCR DNA and Gel Band Purification kit (GE Healthcare, Little Chalfont, Bucks, United Kingdom). An amplicon library was constructed with equimolar concentrations of the amplicons, and emPCR was conducted according to the Rapid Library preparation kit instructions (Roche, Basel, Switzerland). DNA beads were successfully deposited onto the PicoTiterPlate and sequenced with a GS Junior system (Roche).

The *amoA* sequences generated in this study were processed using the microbial ecology community software program, Mothur (Schloss et al., 2009). The sequences were de-noised and the barcode and forward primer sequences were removed simultaneously with the shhh.seqs (sigma value = 0.01) and trim.seqs scripts, and chimeric sequences were identified with chimera.uchime (Schloss et al., 2009). Reads shorter than 400 bp in lengthand sequences containing undetermined nucleotides were removed. The remaining sequences were aligned with the *amoA* DNA sequences from the NCBI nucleotide database, and then any sequences that could not be aligned with the previously discovered *amoA* sequences were removed. The phylogenetic distances between these high quality sequences were calculated with Mothur (Schloss et al., 2009), and operational taxonomic units (OTUs) were generated with 97% DNA sequence similarity as the cutoff value. The OTUs that contained just one sequence were removed. The richness estimator (Chao1), diversity (Shannon–Weaver index, *H*′), and Good’s coverage were calculated with 97% sequence similarity as cutoff values. To evaluate the number of shared OTUs among the samples, a Venn diagram was generated with Mothur (Schloss et al., 2009), using a 97% DNA sequence similarity as the cutoff value. A rarefaction curve was also generated, again with a 97% sequence similarity as the cutoff value. The OTUs with relative abundances > 0.1% of the relative abundance of the whole dataset, were regarded as being the principal (or top) OTUs and these were selected for subsequent analysis. The remaining OTUs were treated as a minor group.

To identify the phylogenetic affiliation of *amoA* sequences, representative sequences of the top OTUs were used to search the nucleotide BLAST (BLASTn) webpage of the NCBI nucleotide sequence database^1^. The representative sequences of the top OTUs, the selected reference sequences and the environmental sequences of the *amoA* gene from the NCBI database were used to construct a Maximum-likelihood (ML) tree using the MEGA 6.0 (molecular evolutionary genetics analysis) software (Tamura et al., 2013). The DNA sequences were codon-aligned and a model test was conducted to select the best fit DNA substitution model for construction of the ML tree. Based on the Bayesian Information Criterion calculation, the Tamura 3-parameter model, using discrete Gamma distribution with the assumption that a certain portion of sites are evolutionarily invariable (T92+G+I), was selected. The ML tree was further edited with iTOL (Letunic and Bork, 2016), with the relative abundances of the top OTUs displayed. To evaluate the number of shared OTUs among samples, the normalized OTU data were also used to generate a Venn diagram with Mothur (Ihaka and Gentleman, 1996).

### Statistical Analysis

To assess the dissimilarity among multiple groups, a newick-formatted tree was generated using the tree.shared command in Mothur, and the Bray–Curtis calculator was used to determine the UPGMA (unweighted pair group method with arithmetic mean) clustering. In addition, Pearson’s correlation coefficients between the environmental variables and the proportions of different clusters, and the abundance of WCA and WCB genes from the different stations were calculated using the SPSS software package (SPSS, Chicago, IL, United States) after the data were square-root transformed. Values of *p* < 0.05 and *p* < 0.01 were considered to indicate different levels of statistical significance.

### Accession Number

All the *amoA* sequences obtained from this study have been deposited in the National Center for Biotechnology Information (NCBI) Sequence Read Archive (SRA) under the accession number of SRP094399.

## Results

### Hydrographic Conditions

**FIGURE 2:**
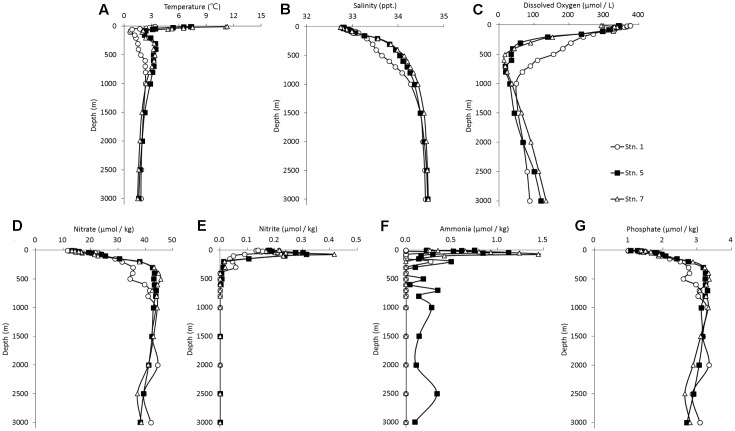
Vertical profile of the hydrographical conditions of the sampling stations in the Sea of Okhotsk and western subarctic Pacific: **(A)** temperature; **(B)** salinity; **(C)** dissolved oxygen; **(D)** nitrate; **(E)** nitrite; **(F)** ammonia; **(G)** phosphate.

Our data showed that the water columns at Stn. 5 and Stn. 7, both of which were located in the northwestern Pacific, exhibited similar hydrographic characteristics (**Figure 2**). The temperature decreased sharply from the surface to subsurface depths, corresponding to a stable thermocline being formed. Concomitantly, the salinity increased noticeably from the surface to the mesopelagic zone, while DO concentration decreased from the surface to a depth of around 500 m when it reached a minimum. The concentrations of nitrate and phosphate appeared to reach a peak at a depth of around 500 m, and these high levels were maintained into the deeper waters. In contrast, the concentrations of nitrite and ammonia reached their maximum at around 50 m, they then dropped sharply to the background value and they were then essentially undetectable along the rest of the vertical profile. Comparatively, the DO concentration was higher in the bathypelagic waters than in the mesopelagic waters, whereas the other environmental factors were similar at these two depths (**Figure 2**). Stn. 1, which is located at the Kuril Basin of the Sea of Okhotsk near the Bussol Strait, showed similar vertical shifting patterns as the other two stations, but much lower values were detected for almost all the factors except for the DO concentration. In the surface water of Stn. 1, the low temperature and high DO recorded are due to the strong turbulent diapycnal mixing in and around the Kuril Straits.

### Diversity and Community Composition of AOA

Pyrosequencing generated ∼5,000 quality reads for each sample, except for those obtained at depths of 5 m at Stn. 5 and Stn. 7 when ∼3,300 and ∼1,800 quality reads, respectively were generated (**Table 1**). On average, >550 bp per read was obtained for each sample. Using a dissimilarity cutoff value of 3%, the highest community diversity (*H*′) of AOA was always shown in the mesopelagic zone (1000 m). The diversity of the AOA communities was highly varied among the three geographic sampling stations. For a comparison purpose, samples collected at depths of 200 m at each station were also investigated at the cDNA level, to reflect these transcriptionally active portions. The results show that the total number of OTUs and the *H*′ were comparable at the DNA and cDNA levels at Stn. 1, whereas both were significantly reduced at the cDNA level at Stns 5 and 7 (**Table 1**). In addition, the coverage values for samples at the three stations all exceeded 97% (**Table 1**). This was consistent with the patterns of the rarefaction curves with cutoff values of 3% (Supplementary Figure S1), which indicate that sufficient sampling efforts were made in order to adequately assess the microbial community composition in each sample under investigation.

**Table 1:** Sequencing statistics and diversity estimates for the samples collected from the different locations in this study.

Stations	Location	Depth (m)	High quality reads	Average length (bp)	97%			


					OTU	Chao	*H′*	Coverage
Stn. 1	47°05***′*** N, 151°00***′*** E	5	4959	619	168	237	2.9	0.99
		200	4986	617	99	184	1.6	0.99
		1000	4959	619	207	249	3.2	0.99
		3000	4944	625	95	132.7	2.4	0.99
		(cDNA) 200	4934	616	105	232.2	2.1	0.99
Stn. 5	45°25*′* N, 153°00*′* E	5	3367	546	160	219.2	2.6	0.98
		200	4991	566	169	355.5	2.4	0.98
		1000	4975	576	157	191.9	3	0.99
		3000	4974	577	90	117	1.9	0.99
		(cDNA) 200	4998	622	55	101.4	1.5	0.99
Stn. 7	44°35*′* N, 154°00*′* E	5	1795	549	71	79.5	3.1	0.99
		200	4988	571	200	306.6	2.4	0.98
		1000	4968	558	285	336.8	3.7	0.98
		3000	4894	538	310	424.1	3.7	0.97
		(cDNA) 200	4999	618	55	97.9	1.7	0.99


**FIGURE 3:**
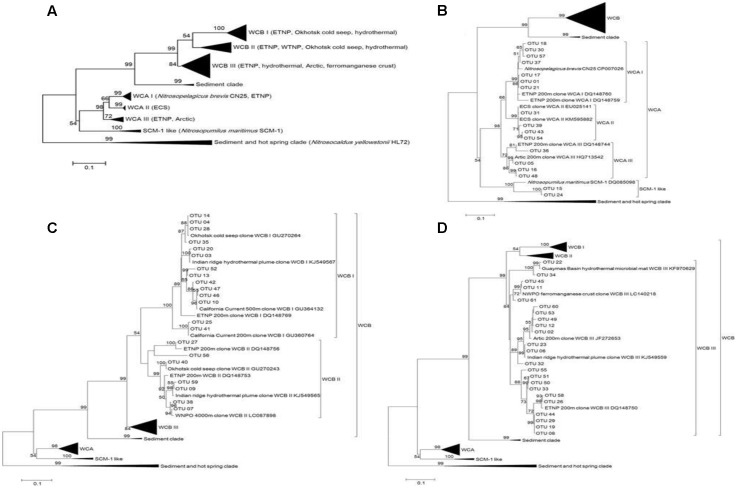
Maximum-likelihood phylogenetic tree illustrating the 61 most abundant OTUs at the different sampling stations in the Sea of Okhotsk and western subarctic Pacific **(A)** and an expanded view for clusters WCA **(B)** and WCB **(C,D)**. A bootstrap value greater than 50% is shown (calculated 1,000 times).

Phylogenetic trees constructed using maximum-likelihood (ML) demonstrated that the 61 most abundant OTUs fell into three clusters (**Figure 3**). These were the estuary coast and shallow water source cluster [water column A (WCA)] (15 OTUs), the deep sea source cluster [water column B (WCB)] (44 OTUs) and the SCM-1-like AOA cluster (2 OTUs) (**Figure 3A**). Three sub-clusters were formed in the WCA cluster: WCAI (7 OTUs); WCAII (3 OTUs); and WCAIII (4 OTUs) (**Figure 3B**). WCB consisted of three sub-clusters, namely: WCBI (14 OTUs); WCBII (7 OTUs) (**Figure 3C**); and WCBIII (23 OTUs) (**Figure 3D**).

**FIGURE 4:**
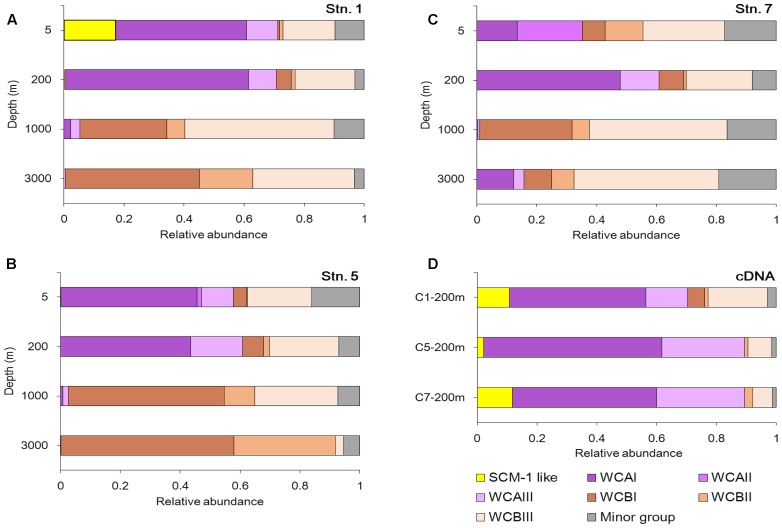
Community composition of AOA at the DNA level **(A–C)** and the cDNA level **(D)** at the different sampling stations in the Sea of Okhotsk and western subarctic Pacific.

Almost all the sub-clusters were detected across different water depths at the three stations (**Figures 4A–C**). The SCM-1-like cluster, closely related to the widely distributed *N. maritimus* group essentially found in all open oceans worldwide, was mainly detected in the surface water in our study, and exhibited its highest relative abundance at Stn. 1 (**Figure 4A**). WCA is normally well-adapted to shallow-water, and in our study its maximum was detected in the upper ocean (5 and 200 m). WCAI was the second most abundant sub-cluster at 3000 m at Stn. 7 (**Figure 4C**), which makes this sample relatively distinct from the other deep water samples. WCAII was detected mainly at a depth of 5 m at Stn. 7 (**Figure 4C**), and this contributed to the differentiation from the other euphotic samples. WCBI and WCBII seemed to be more specific in the deep waters, especially at Stns 1 and 5 (Supplementary Figure S2). WCBIII was the largest cluster within WCB and contributed significantly to the AOA community in the euphotic and deep waters. The vertical distribution pattern of WCAI was opposite to that of WCBI, showing clear successions from the euphotic zone to deep water.

At the cDNA level, a similar community composition of AOA was shown at the three stations (**Figure 4D**). However, spatial variations in the community compositions of AOA were clearly shown between the DNA and cDNA levels. For example, with the current sequencing depth, the SCM-1 like cluster was too rare to be detected at the DNA level at 200 m, but its portion at the cDNA level was comparable with that of the WCA cluster. In addition, the relative abundance of WCB in the cDNA samples was lower than in their corresponding DNA samples.

### Abundance of AOA

**FIGURE 5:**
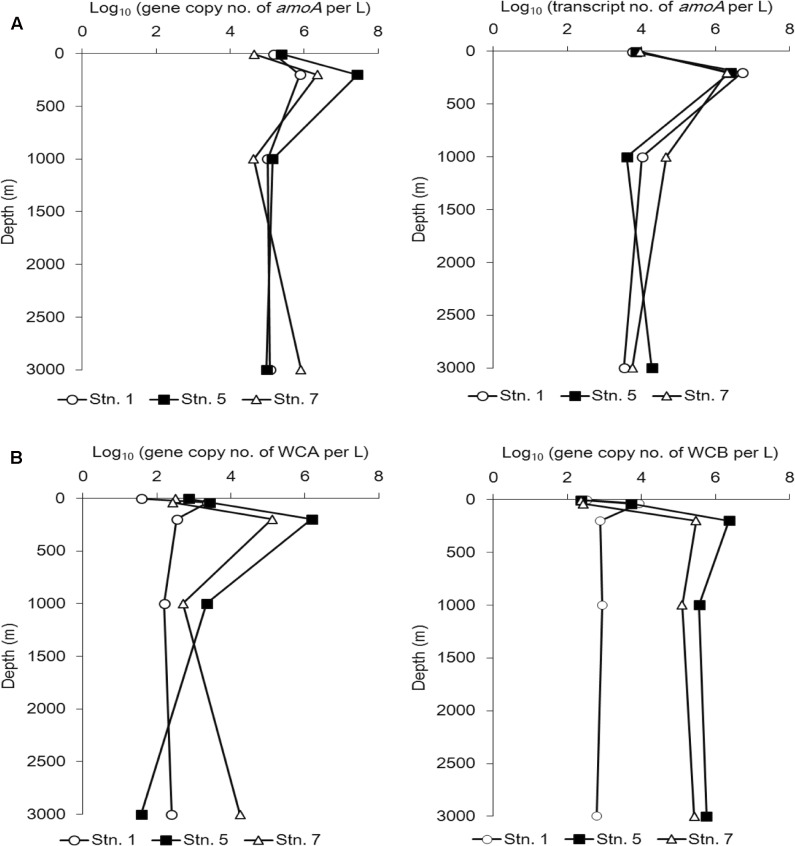
Abundance of the *amoA* gene at the DNA level (**A**, left) and cDNA level (**A**, right) and the WCA (**B**, left) and WCB (**B**, right) genes at different sampling stations in the Sea of Okhotsk and western subarctic Pacific.

The *amoA* gene and gene transcript abundance reached its maximum at 200 m at all three stations (**Figure 5A**).

With the exception of Stn. 1, the abundance of this gene at 200 water depth was always higher than that of the gene transcript (**Figure 5A**).

We also quantified the two main AOA clusters along the vertical profile at the three stations in further detail (**Figure 5B**). Although the abundance of WCA was obviously reduced in the bathypelagic water (3000 m) at Stn. 5, on the whole the abundance of WCA and WCB demonstrated a similar vertical distribution pattern at Stn. 5 and Stn. 7. Thus, at both stations, the lowest abundance of WCA and WCB was found at the surface, they reached a maximum level at 200 m, and then decreased in the deep waters (1000 and 3000 m), although WCB was more abundant than WCA in the mesopelagic and bathypelagic waters (**Figure 5B**).

### Correlation with Environmental Parameters

The distribution of AOA phylogenetic sub-clusters were significantly correlated with a number of the water-column properties (**Table 2**). For example, the SCM-1-like cluster was negatively correlated to PO_4_ (*r* = -0.62) and NO_3_ (*r* = -0.57), but positively correlated to DO (*r* = 0.60); WCAII was significantly influenced by temperature and the concentration of NO_3_ and NO_2_; whereas WCAIII was only affected significantly by the depth (*r* = -0.57).

**Table 2:** Pearson correlation coefficients between the environmental variables obtained from the sampling stations and the proportions of different sub-clusters (the first eight rows retrieved from pyrosequencing), and the abundance of the WCA and WCB genes (the last two rows quantified by qPCR) from this study.

	Salinity	Depth	Temperature	DO	NO_3_	NO_2_	NH_4_	PO_4_	Si
SCM-1-like	-0.45	-0.27	-0.02	0.60ˆ*	-0.57ˆ*	0.37	-0.15	-0.62ˆ*	-0.41
WCAI	-0.78ˆ**	-0.69ˆ*	0.17	0.71ˆ**	-0.56	0.44	0.47	-0.55	-0.80ˆ**
WCAII	-0.53	-0.34	0.94ˆ**	0.44	-0.60ˆ*	0.78ˆ**	0.25	-0.54	-0.55
WCAIII	-0.48	-0.57ˆ*	-0.18	0.37	-0.20	0.05	-0.44	-0.22	-0.49
WCBI	0.81ˆ**	0.65ˆ*	-0.34	-0.80ˆ**	0.70ˆ**	-0.61ˆ*	-0.36	0.69ˆ*	0.84ˆ**
WCBII	0.62ˆ*	0.75ˆ**	-0.06	-0.44	0.34	-0.27	-0.42	0.31	0.61ˆ*
WCBIII	0.25	0.03	-0.03	-0.37	0.32	-0.17	-0.15	0.37	0.25
WCA gene copy^#^	0.01	-0.16	-0.15	-0.17	0.27	-0.18	-0.12	0.26	-0.02
WCB gene copy^#^	0.55ˆ*	0.34	-0.49	-0.59ˆ*	0.65ˆ**	-0.58ˆ*	-0.26	0.61ˆ*	0.54ˆ*


^#^*n* = 15 and for all others *n* = 12; significance ^∗^*p* < 0.05; ^∗∗^*p* < 0.01.

WCBII was affected by the depth, salinity, and Si; however, none of the environmental variables had significant influence on the distribution of WCBIII. The gene abundance of WCB was positively affected by salinity and the concentration of NO_3_, PO_4_, and Si. In contrast, no significant correlations were found between the WCA gene abundance and any of the environmental variables measured.

### Community Similarity

**FIGURE 6:**
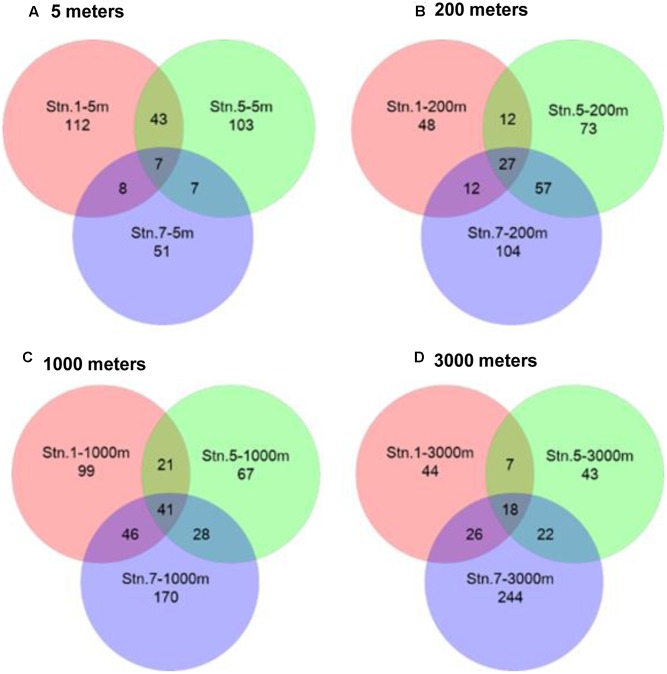
Venn diagrams representing the overlap of OTUs among different sampling stations in the Sea of Okhotsk and western subarctic Pacific. **(A)** 5 m; **(B)** 200 m; **(C)** 1000 m; **(D)** 3000 m.

**FIGURE 7:**
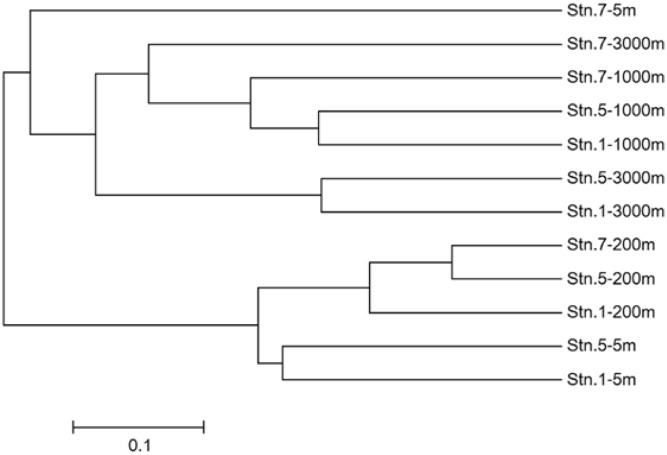
Unweighted pair group method with arithmetic mean (UPGMA) clustering based on total OTUs from different sampling stations in the Sea of Okhotsk and western subarctic Pacific. The Bray–Curtis similarity was used for clustering analysis.

Venn diagrams showed the overlapping of OTUs among samples (**Figure 6**). Stn. 1 and Stn. 5 had the highest number of shared OTUs (43 OTUs) in the surface waters. The highest number of OTUs (41 OTUs) shared by the three stations was detected at 1000 m; while the highest number of specific OTUs occurred in the bathypelagic waters at Stn. 7 (158 OTUs) (Supplementary Figure S3). This OTU distribution pattern among the three oceanic stations explains the UPGMA clustering dendrogram (**Figure 7**), which demonstrates that samples from the surface and subsurface layers (5 and 200 m) were closely clustered together and clearly separated from the samples collected in the deep waters (1000 and 3000 m).

## Discussion

### Abundance of *amoA* Gene and Gene Transcript

Our *amoA* gene abundances are comparable to those detected in the Atlantic Ocean (Agogué et al., 2008). Low AOA abundance appeared in the upper euphotic zone might be caused by a low growth rate of AOA, either inhibited by light (Merbt et al., 2012) or competed with phytoplankton for ammonium uptake (Smith et al., 2014); while in deep bathypelagic waters might due to the low availability of ammonia (<10 nM) beyond the detection limit of our method.

So far, most AOA quantification studies have been conducted at the DNA level (Agogué et al., 2008; Beman et al., 2012; Peng et al., 2013), but the discrepancies between the abundance of *amoA* gene and gene transcript were revealed in recent studies (Church et al., 2010; Smith et al., 2016). In our study, both *amoA* gene and gene transcript peaked at 200 m; this is in agreement with their maxima appeared at the base of the euphotic zone in the Atlantic Ocean (Agogué et al., 2008). The N maximum at the base of the euphotic zone resulted from the remineralization of sinking organic materials might be a reason for the high gene transcript occurred (Beman et al., 2012). The higher abundance of *amoA* gene transcript at Stn.1 (located in the marginal sea), as compared with that at the two stations located in the Pacific Ocean may be related to terrestrial input or a strong mixing occurred at 200 m indicated by the relatively high concentrations of ammonia and nitrite. In addition, the first quantification of WCA and WCB clusters was conducted only recently in the central California Current (Santoro et al., 2013), where exhibited a comparable abundance of our WCA but almost undetectable amounts of WCB, possibly due to the shallow sampling depths used (≤500 m).

### Variations of Diversity, Phylogeny, and Composition of AOA

The highest diversity of AOA in the mesopelagic zone (1000 m) might be due to that the higher levels of light in the euphotic zone inhibits the AOA, resulting in a decrease in the community diversity of AOA (Merbt et al., 2012). Significantly reduced total number of OTUs and *H*′ at the cDNA level at Stns 5 and 7 suggests that only a small portion of the AOA might be transcribing *amoA* gene, since not all the AOA communities are actively involved in ammonia oxidization (Church et al., 2010; Smith et al., 2014).

The phylogeny of AOA in this study was in agreement with previous reports, which showed that marine AOA are generally predominated by the phylogenetically distinct shallow WCA (< 200 m depth) and deep WCB (>200 m depth) clusters (Francis et al., 2005; Beman et al., 2008). In our study, both WCBI and WCBII were represented by sequences from the cold seep of the Okhotsk Sea, the hydrothermal plume and the eastern tropical North Pacific Ocean (ETNP); whereas sequences in WCBIII were affiliated to those from the bathypelagic hydrothermal vent (Winkel et al., 2014), the ETNP (Francis et al., 2005) and the Arctic (Pedneault et al., 2014). The habitat-specific groupings observed for the different WCB sub-clusters are thought to reflect the selective conditions of the habitats and the influences of the spatial separation on the occurrence of isolated microevolution.

The most abundant OTU01 that is closely related to the ubiquitous *Nitrosopelagicus brevis* (Santoro et al., 2015) presented throughout the water column, indicating its flexibility to adapt to a range of different hydrographic conditions. It should be noted that WCAII has previously been reported to be specific to the East China Sea (Hu et al., 2011), and it has not been reported at the East side of the Pacific Ocean; therefore, this sub-cluster is likely distributed mainly along the marginal seas of the West Pacific. On the other hand, WCBI and WCBII both increased from the euphotic zone to the deeper waters, containing genotypes closely clustered with those previously reported from the Okhotsk Sea cold seep (Dang et al., 2010). In addition, at the cDNA level, it was not surprising to find that WCA (I and III) was predominant in all the stations, because all the cDNA samples were collected from 200 m, where the environmental condition would be suitable for the shallow-water-adapted WCA rather than WCB.

### Environmental Impacts and Community Similarity

The composition and distribution of the AOA community have been reported to be influenced by diverse environmental factors (Erguder et al., 2009). AOA *amoA* gene abundances were essentially determined by ammonia concentration (Christman et al., 2011), and were correlated with NO_2_/NO_3_ maxima in the oceans as well (Mincer et al., 2007; Beman et al., 2008). By far, most studies have only measured the environmental effects on the total AOA/AOB or WCA/WCB communities, rather than extending to the level of the phylogenetic sub-clusters. The positive correlation of WCBI and WCBII to the depth, and negative correlation of WCBI to the DO further supports the general preferential distribution of WCB (Molina et al., 2010).

The clear zonation of the community clusters in the euphotic zone and deep waters indicates that AOA have little vertical exchange and they have specific distribution. Indeed, AOA species have never been reported to be mobile, and no vertical fluxing occurred in our sampling stations. The separation of AOA in the deeper waters (1000–3000 m) from shallower water depths supports that microbial communities in the surface/subsurface waters are distinct from those in the deeper waters even in geographically distant locations (Jing et al., 2013). The surface water (5 m) at Stn. 7 was distinct from those at the other two stations, which were located in the Sea of Okhotsk and Oyashio Current, and a horizontal transport of water might occur in between. In addition, the habitat-specific groupings in the different WCB sub-clusters were observed, reflecting the occurrence of isolated microevolution due to spatial separation. Our data therefore showed stratification of AOA in the water column and a biogeographically distinct distribution pattern in the western subarctic Pacific, which might be attributed to selective pressure due to the different *in situ* physical/chemical conditions.

Studies on the Arabian Sea and the Eastern Tropical South Pacific Ocean demonstrate that geographical variation exerts a strong control over the AOA community structures (Peng et al., 2013). In our study, the AOA communities exhibited a clear adaptation of the “ocean water column” and the effect of geographic separation by the Kuril Islands is not as clear as expected. We hypothesize that diapycnal mixing associated with strong tidal currents in and around the Kuril Straits, particularly the water exchange through the Bussol Strait would result to a certain extent in an almost homogenous AOA community structure. In order to test this hypothesis, however, other chemical/physical oceanographic data are required.

We initially also attempted to study the community composition and abundance of AOB, but its *amoA* gene could not be successfully amplified. This is in many ways not surprising as AOB has been reported to be undetectable by qPCR and pyrosequencing in various other locations, including in the Gulf of California (GOC) (Beman et al., 2008), the Atlantic Ocean (Wuchter et al., 2006) and the ETNP (Stewart et al., 2012), and including at depths and stations where ammonia oxidation rates were substantial and AOA were detected. However, AOB are likely to be actively competing with AOA for a common substrate and for ecological niches and they have been observed in the deep ocean (2000 and 2956 m) in the Northeastern Japan sea (Nakagawa et al., 2007), and so their role in nitrogen cycling in the northwestern Pacific Ocean should not be ignored. Additional studies are needed to discern the specific oceanic conditions under which AOB are more abundant and diverse. In addition, while PCR-based studies are an essential part of AOA research, incorporating these together with isotopic labeling techniques for *in situ* rate measurements, “meta-omics”/whole-genome sequencing approaches; and the physiological characterization would facilitate a deeper understanding of AOA in terms of their additional function, activity and niche segregation in the marine biogeochemical N-cycle.

## Author Contributions

Conceived and designed the experiment: HL and HJ; performed the experiment: SC and XX; analyzed the data: SC, KS, and JN; contributed reagents/materials/analysis tools: HL and HJ; wrote the paper: HJ, HL, and SC.

## Conflict of Interest Statement

The authors declare that the research was conducted in the absence of any commercial or financial relationships that could be construed as a potential conflict of interest.

## Footnotes

1 http://blast.ncbi.nlm.nih.gov/Blast.cgi
