# Supplementary material for: Geographic Distribution of Ammonia-Oxidizing Archaea along the Kuril Islands in the Western Subarctic Pacific
Source: Front Microbiol. 2017 Jun 30;8:1247. doi: 10.3389/fmicb.2017.01247 (PMC5492448; doi:10.3389/fmicb.2017.01247)
Supplement: Supplementary file 1 [file Data_Sheet_1.docx]

**Supplementary materials:**

**Figure S1:** Rarefaction curves of the *amoA* gene sequences obtained from different sampling stations in the Sea of Okhotsk and western subarctic Pacific with 97% sequence similarity as cutoff value.

**Figure S2:** Phylogeny and distribution of the most abundant 61 OTUs among the different sampling stations in the Sea of Okhotsk and western subarctic Pacific.

**Figure S3:** Venn diagrams representing the overlap of OTUs among different water depths of each sampling station in the Sea of Okhotsk and western subarctic Pacific.

**Figure S1**

**
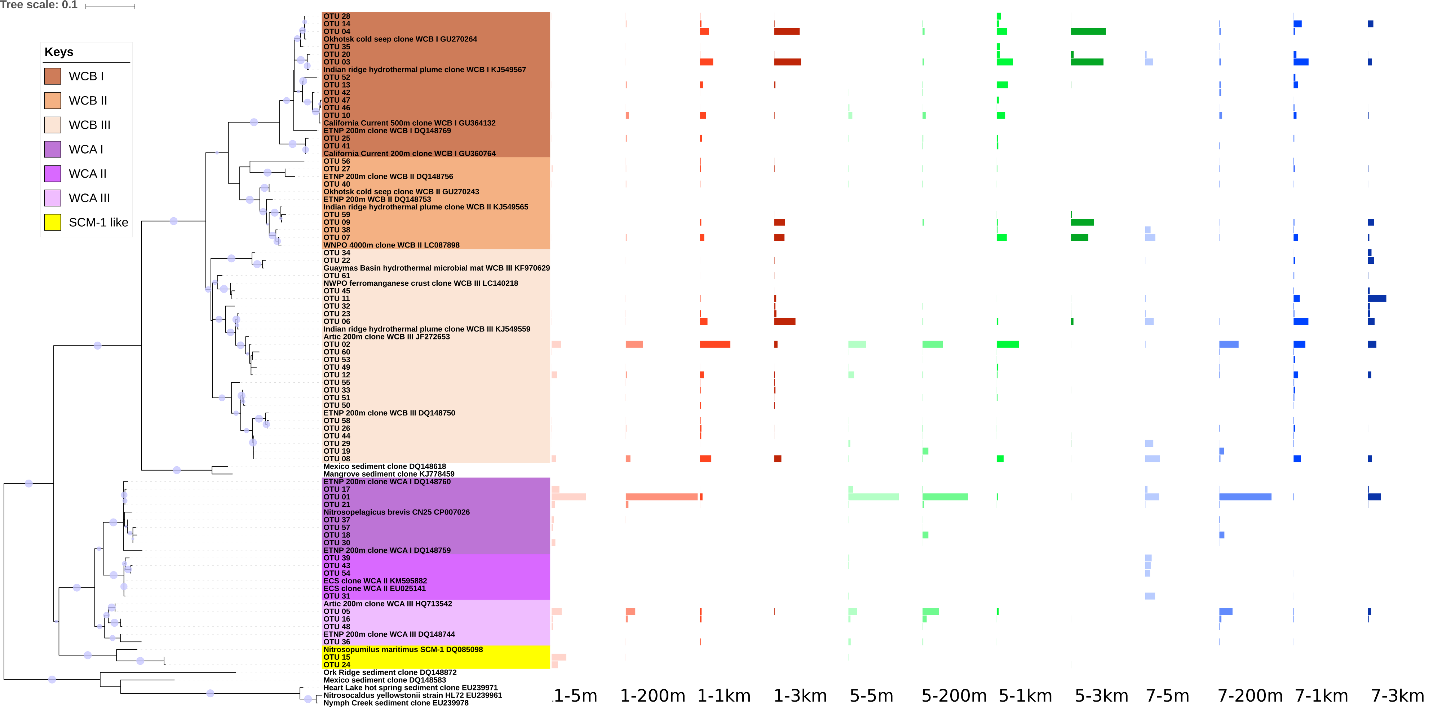
**

**Figure S2**

**Figure S3**
